# A large-scale population-based epidemiological study on the prevalence of central sensitization syndromes in Japan

**DOI:** 10.1038/s41598-021-02678-1

**Published:** 2021-12-02

**Authors:** Yasuo Haruyama, Toshimi Sairenchi, Koji Uchiyama, Keisuke Suzuki, Koichi Hirata, Gen Kobashi

**Affiliations:** 1 Integrated Research Faculty for Advanced Medical Sciences, Dokkyo Medical University, 880 Kitakobayashi, Mibu, Shimotsuga, Tochigi, 321-0293 Japan; 2 Department of Public Health, Dokkyo Medical University School of Medicine, Tochigi, 321-0293 Japan; 3 Laboratory of International Environmental Health, Center for International Cooperation, Dokkyo Medical University, Tochigi, 321-0293 Japan; 4 Department of Neurology, Dokkyo Medical University, Tochigi, 321-0293 Japan

**Keywords:** Pain, Chronic pain, Epidemiology, Neurological disorders, Psychiatric disorders

## Abstract

A cross-sectional study of 21,665 Japanese residents was performed to investigate the prevalence of central sensitization syndromes (CSS). CSS were assessed using the Central Sensitization Inventory (CSI-A). CSS were defined as a CSI-A score of 40 or higher. Age, sex, district, 10 CSS-related diseases (CSI-B), lifestyle, and mental factors were rated in a self-reported survey. The prevalence of CSS and its relationship with potential factors were examined by sex using descriptive and logistic regression models. The prevalence of CSS was 4.2% in all participants and was significantly higher in women (4.9%) than in men (2.7%). Adjusted odds ratios correlated with CSS for an age of 80–97 years versus 60–79 years (2.07 and 2.89), one or more CSI-B diseases (3.58 and 3.51), few sleeping hours (2.18 and 1.98), high perceived stress (5.00 and 4.91), low (2.94 and 2.71) and high (0.45 and 0.66) resilience versus moderate resilience, and exercise habits (0.68 and 0.55) in men and women (all *P* < 0.05). The relationship between CSS and age 20 and 59 years, ex-smokers, coffee intake, and alcohol intake differed by sex. The prevalence of CSS was estimated to be low in the healthy population. CSS correlated with CSS-related diseases and some positive and negative factors.

## Introduction

In clinical medicine, central sensitization syndromes (CSS) are overlapping disorders of multiple psychoneurological, musculoskeletal, and chronic pain-related diseases^1–3^. CSS mainly include fibromyalgia (FM), restless leg syndrome (RLS), chronic fatigue syndrome (CFS), temporomandibular joint disorder (TJD), migraine or tension-type headaches (M/TTH), irritable bowel syndrome (IBS), multiple chemical sensitivity (MCS), and posttraumatic stress disorder (PTSD)^1,2,4,5^. CSS are characterized by complex physical and mental symptoms, mainly disproportionate pain and diffuse pain distribution manifested by hypersensitivity of the central neurons, which have a negative impact on the quality of life of patients^1,2,6^. CSS are intractable and there are currently no established medical interventions due to the lack of a clinically obvious pathology^4,7^.

In 2012, Mayer et al. initially developed Central Sensitization Inventory (CSI) part A, comprising 25 symptoms, and part B, consisting of 10 diseases, to evaluate CSS^8^. A 40-point cut-off score of CSI part A was recommended to classify the presence of CSS in patients with chronic pain^6,8^. Many clinical studies have since reported the rates of a CSI score ≥ 40 in patients with CSS-related diseases: 11.0% in 290 orthopedic patients with musculoskeletal pain, including the neck, shoulders, hips, knees, or ankles^9^, 13% in 238 patients with low back pain^10^, 34.9% in 66 patients with pelvic floor muscle tenderness^11^, 48.4% in 91 patients with osteoarthritis^12^, 68.2% in 763 patients with chronic spinal pain disorder^13^, and 84% in 32 patients with hereditary neuropathy^14^. These findings demonstrated that the prevalence of CSS varied in a CSS-related disease-dependent manner; however, data on the general population is lacking.

Neblett et al. previously reported that the rate of a CSI score ≥ 40 was 24.8% in 129 non-patient subjects, with an average CSI score (SD) of 30.9 (12.3)^15,16^. Other studies found average CSI scores (SD) of 37.1 (15.0) in 63 healthy individuals^17^, 28.9 (13.5) in 40 university students^8^, and 16.2 (11.8) in 20 healthy individuals^18^. Only a few studies have examined healthy individuals as a non-patient comparison sample; however, these sample sizes were small and did not avoid selection bias. To the best of our knowledge, an epidemiological study has not yet been conducted, and, thus, the rate of a CSI score ≥ 40 in the general population remains unclear. Therefore, we herein investigated the prevalence of CSS using a large-scale general population-based survey.

## Results

### Descriptive statistics of CSS and each variable

**Figure 1:**
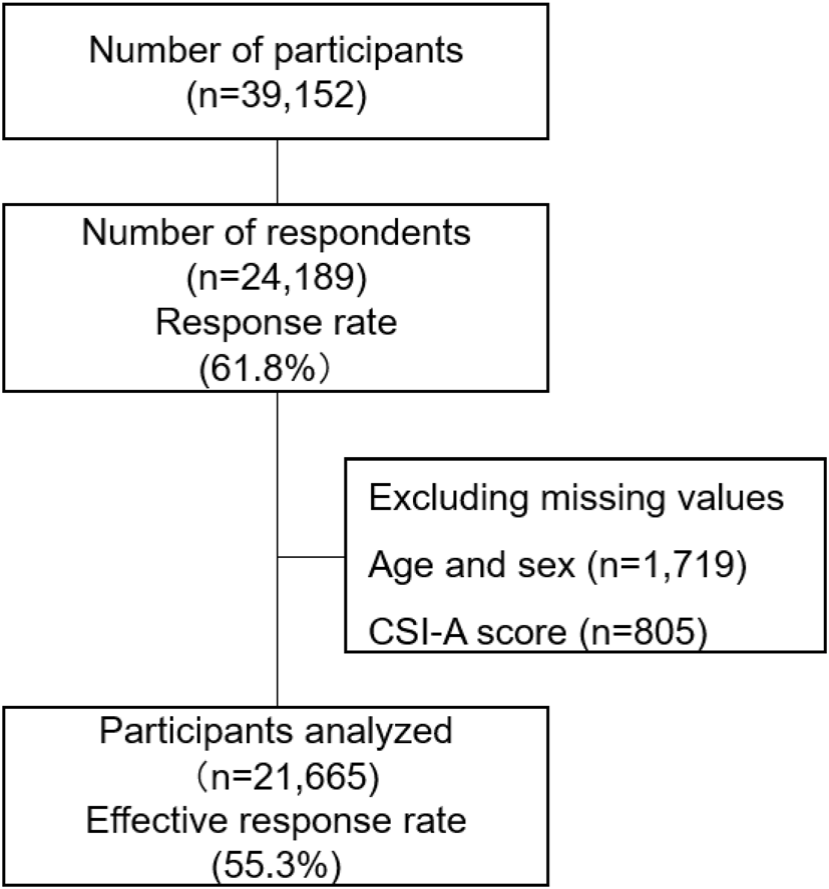
Chart of the epidemiological survey. *CSI* Central sensitization inventory.

Following the exclusion of participants with missing values, including age and sex (n = 1719) as well as CSI (n = 805), 21,665 were ultimately analyzed in the present study. The effective response rate was 55.3% (Fig. 1). Table 1 shows the characteristics of participants. The average age (SD) of participants was 63.4 yr. (11.7) for all, 66.6 yr. (10.8) for men, and 61.7 yr. (11.8) for women. The rate of depression was the highest among the 10 CSS-related diseases in CSI-B, while none of the participants reported TJD or MCS. In total, 3761 (17.4%) and 1522 (7.0%) participants were missing values on coffee and alcohol intake, respectively.

**Table 1:** Characteristics of participants by sex.

		Total (n = 21,665)		Men (n = 7527)		Women (n = 14,138)	
		n	%	n	%	n	%
Age, mean (SD), min, max, yr		63.4 (11.7)	20, 97	66.6 (10.8)	25, 96	61.7 (11.8)	20, 97
Age groups, yr	20–39	386	1.8	66	0.9	320	2.3
	40–59	6277	29.0	1479	19.6	4798	33.9
	60–79	14,026	64.7	5432	72.2	8594	60.8
	80–97	976	4.5	550	7.3	426	3.0
District	Rural area	2600	12.0	1209	16.1	1391	9.8
	Urban area	19,065	88.0	6318	83.9	12,747	90.2
CSI-B-related diseases, yes							
Migraine or tension headaches		18	0.08	4	0.05	14	0.10
Restless legs syndrome		3	0.01	1	0.01	2	0.01
Chronic fatigue syndrome		2	0.01	0	0.00	2	0.01
Fibromyalgia		2	0.01	0	0.00	2	0.01
Temporomandibular joint disorder		0	0.00	0	0.00	0	0.00
Irritable bowel syndrome		14	0.06	5	0.07	9	0.06
Multiple chemical sensitivities		0	0.00	0	0.00	0	0.00
Neck injury (including whiplash)		3	0.01	1	0.01	2	0.01
Anxiety or panic attacks		53	0.24	7	0.09	46	0.33
Depression		170	0.78	57	0.76	113	0.80
Smoking^a^	Non-smoker	14,778	69.9	2734	37.9	12,054	86.4
	Ex-smoker	4853	22.9	3492	48.4	1361	9.7
	Smoker	1530	7.2	986	13.7	544	3.9
Alcohol intake^a^	Non-drinker	11,514	57.2	2426	37.3	9088	66.7
	≤ 1 day per week	2969	14.7	904	13.9	2,065	15.1
	> 1 day per week	5660	28.1	3180	48.8	2480	18.2
Coffee intake^a^	Non-drinker	2506	14.0	991	16.0	1515	12.9
	≤ 1 day per week	2693	15.0	941	15.1	1752	15.0
	> 1 day per week	12,705	71.0	4279	68.9	8426	72.1
Exercise habits^a^	No	10,129	47.8	3315	44.8	6814	49.4
	Yes	11,078	52.2	4088	55.2	6990	50.6
Sleeping hours per day^a^	≤ 5 h	4459	20.6	1227	16.3	3232	23.0
	6–9 h	17,081	79.1	6250	83.2	10,831	76.9
	≥ 10 h	56	0.3	37	0.5	19	0.1
Perceived stress^a^	Low	12,731	59.3	5373	71.9	7358	52.6
	High	8736	40.7	2102	28.1	6634	47.4
Resilience^a^	Low	3846	17.9	1182	15.8	2661	19.0
	Moderate	8593	39.9	3161	42.2	5432	38.6
	High	9106	42.2	3140	42.0	5966	42.4

*CSI* Central sensitization inventory.

^a^Participants with missing values for smoking (n = 494), alcohol intake (n = 1522), coffee intake (n = 3761), exercise habits (n = 458), sleeping hours per day (n = 69), perceived stress (n = 198), and resilience (n = 120) were excluded.

**Figure 2:**
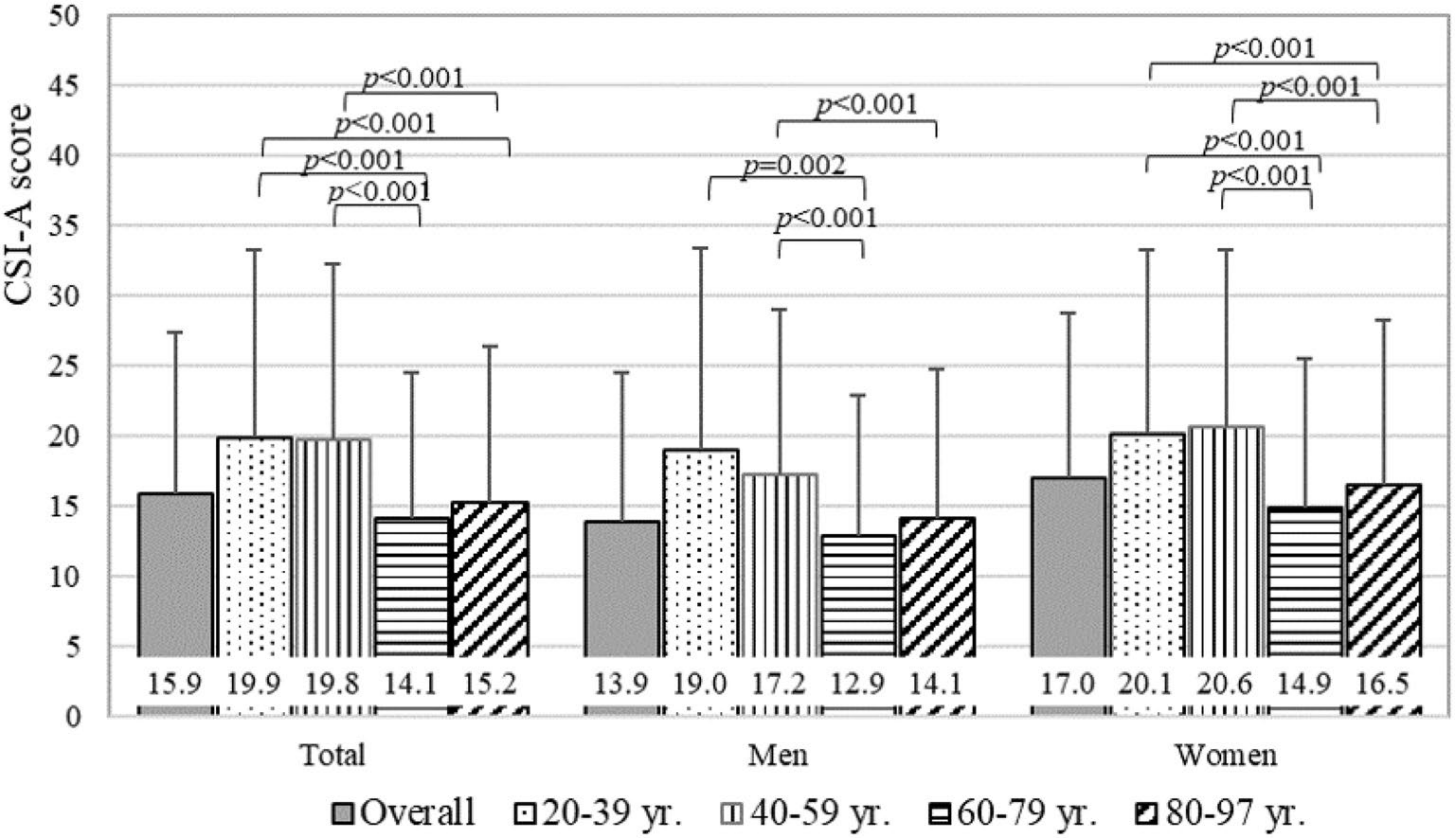
Average CSI-A scores by sex and age groups. *CSS* Central sensitization syndromes, *CSI* Central sensitization inventory. A Kruskal–Wallis test with the Bonferroni post-hoc test was performed among age groups. Error bars indicate the standard deviation, which based on overall and age groups are 11.5, 13.3, 12.5, 10.4, and 11.2 for total, 10.6, 14.4, 11.8, 10.0, and 10.6 for men, 11.8, 13.1, 12.6, 10.6, and 11.8 for women.

**Figure 3:**
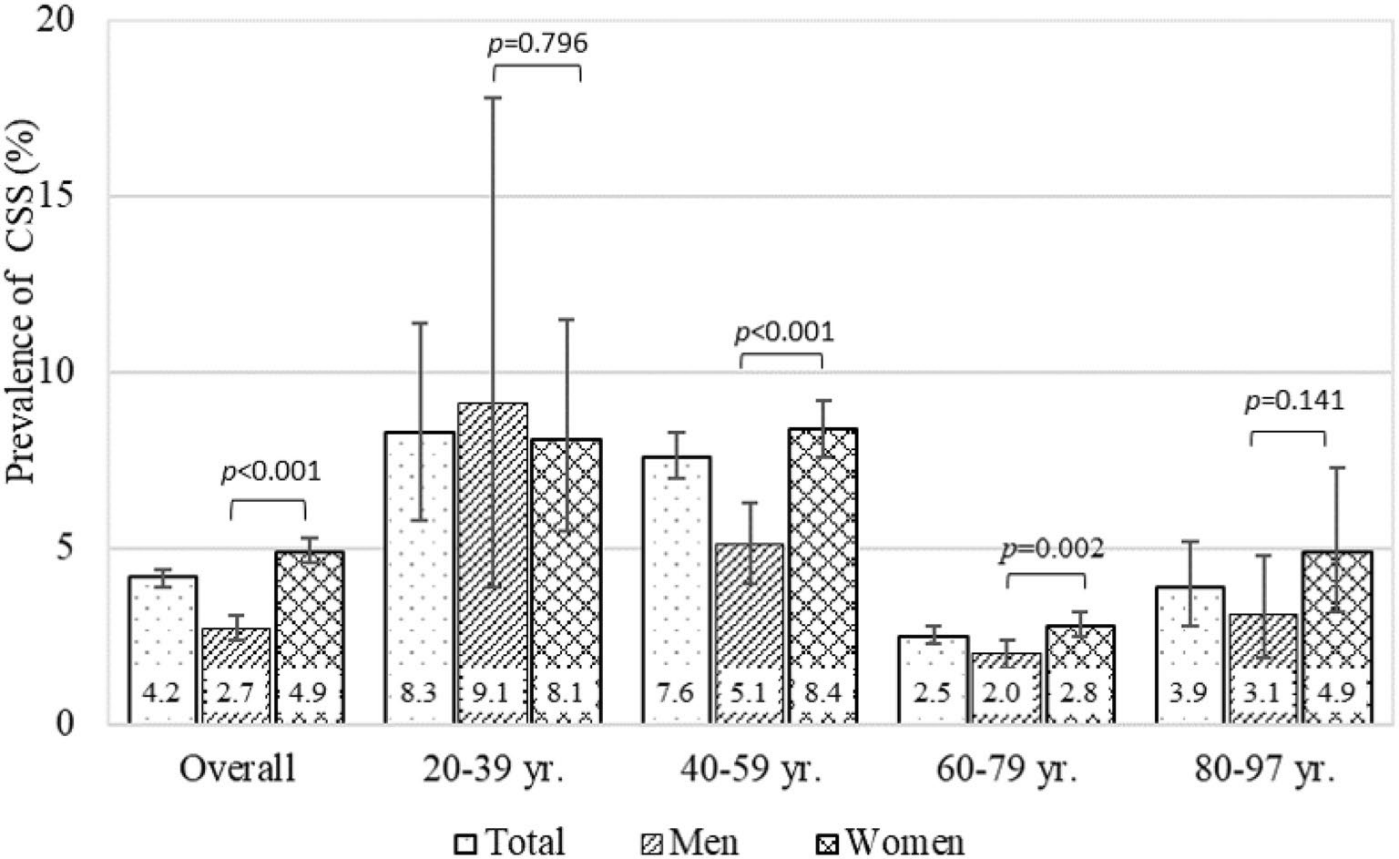
Prevalence of CSS with a CSI-A score ≥ 40 points by sex and age groups. *CSS* Central sensitization syndromes, *CSI* Central sensitization inventory. A chi-squared test was performed between men and women. Error bars indicate the 95% confidence interval, which based on total, men, and women are 3.9–4.4, 2.4–3.1, and 4.6–5.3 for overall, 5.8–11.4, 3.9–17.8, and 5.5–11.5 for 20–39 yr., 7.0–8.3, 4.0–6.3, and 17.6–9.2 for 40–59 yr. 2.3–2.8, 1.6–2.4, and 2.5–3.2 for 60–79 yr., 12.8–5.2, 1.9–4.8 and 3.2–7.3 for 80–97 yr.

Cronbach’s coefficient alpha of CSI-A was 0.893 overall, 0.889 for men, and 0.893 for women. Minimum and maximum CSI-A scores ranged between 0 and 87 points. The average CSI-A score (SD) was 15.9 (11.5) in total and was significantly higher in women [17.0 (11.8)] than in men [13.9 (10.6)] (*P* < 0.001). Among the 4 age groups examined, the average CSI-A score in the 60–79 yr. age group was the lowest (Fig. 2). The prevalence of CSS with a CSI-A score ≥ 40 points in all participants was 4.2% (95% CI 3.9 to 4.4) (Fig. 3). The prevalence of CSS significantly differed between men (2.7%, 95% CI 2.4 to 3.1) and women (4.9%, 95% CI 4.6 to 5.8) (*P* < 0.001). Furthermore, a significant sex difference was observed between the 40–59 yr. and 60–79 yr. groups.

### Relationships between CSS and related factors

CSS with a CSI-A score ≥ 40 points correlated with age groups, CSI-B-related diseases, alcohol intake, exercise habits, sleeping hours per day, perceived stress, and resilience in men and women. In women, smoking and coffee intake correlated with CSS (Table 2).

**Table 2:** Prevalence of CSS with CSI-A ≥ 40 points in each variable by sex.

	CSS with CSI-A ≥ 40 points					
	Men (n = 7527)			Women (n = 14,138)		
	n	%	*P* value^a^	n	%	*P* value^a^
**Age, yr**						
20–39	6	9.1	< 0.001	26	8.1	< 0.001
40–59	75	5.1		403	8.4	
60–79	108	2.0		244	2.8	
80–97	17	3.1		21	4.9	
**District**						
Rural area	31	2.6	0.688	56	4.0	0.108
Urban area	175	2.8		638	5.0	
**CIS-B-related diseases**						
0 diseases	189	2.5	< 0.001	642	4.6	< 0.001
1 disease	17	23.9		51	28.7	
2 diseases	0	0.0		1	16.7	
**Smoking**^**b**^						
Non-smoker	73	2.7	0.628	562	4.4	< 0.001
Ex-smoker	87	2.5		109	8.0	
Smoker	30	3.0		44	8.1	
**Alcohol intake**^**b**^						
Non-drinker	94	3.9	0.001	435	4.8	0.018
≤ 1 day per week	26	2.9		128	6.2	
> 1 day per week	66	2.1		114	4.6	
**Coffee intake**^**b**^						
Non-drinker	24	2.4	0.471	98	6.5	0.018
≤ 1 day per week	31	3.3		88	5.0	
> 1 day per week	115	2.7		400	4.7	
**Exercise habits**^**b**^						
No	130	3.9	< 0.001	471	6.9	< 0.001
Yes	74	1.8		212	3.0	
**Sleeping hours per day**^**b**^						
≤ 5 h	75	6.1	< 0.001	288	8.9	< 0.001
6–9 h	129	2.1		401	3.7	
≥ 10 h	2	5.4		1	5.3	
**Perceived stress**^**b**^						
Low	49	0.9	< 0.001	86	1.2	< 0.001
High	157	7.5		602	9.1	
**Resilience**^**b**^						
Low	102	8.6	< 0.001	331	12.4	< 0.001
Moderate	68	2.2		214	3.9	
High	35	1.1		148	2.5	

*CSS* Central sensitization syndrome, *CSI* Central sensitization inventory.

^a^Using the chi-squared test or Fisher’s exact test.

^b^Participants with missing values for smoking (n = 494), alcohol intake (n = 1522), coffee intake (n = 3761), exercise habits (n = 458), sleeping hours per day (n = 69), perceived stress (n = 198), and resilience (n = 120) were excluded.

Age (20–39 yr. and 40–59 yr. vs 60–79 yr.), CSI-B-related diseases (1 or 2 vs 0 disease), sleeping hours per day (≤ 5 h. vs 6–9 h.), perceived stress (high vs low), and resilience (low vs moderate) showed significantly higher crude odds ratio (cOR), while exercise habits (yes vs no) and resilience (high vs moderate) had significantly lower cOR in both men and women. Alcohol intake (> 1 day per week vs non-drinkers) in men and coffee intake (> 1 day per week vs non-drinkers) in women showed significantly lower cOR, and alcohol intake (> 1 day per week vs non-drinkers) and smoking (ex-smokers and smokers vs non-smokers) had significantly higher cOR in women (Table 3).

**Table 3:** Relationships between the prevalence of CSS with CSI-A ≥ 40 points and factors by sex.

	Men				Women			
	cOR^a^	95%CI	aOR^a^	95%CI	cOR^a^	95%CI	aOR^a^	95%CI
**Age, yr**								
20–39	4.93	2.09–11.66	3.13	1.07–9.19	3.03	1.99–4.61	1.61	0.99–2.62
40–59	2.63	1.95–3.56	1.18	0.80–1.74	3.14	2.67–3.69	1.95	1.59–2.38
60–79	1.00		1.00		1.00		1.00	
80–97	1.57	0.94–2.64	2.07	1.06–4.02	1.77	1.12–2.80	2.89	1.70–4.94
**District**								
Rural area	1.00		1.00		1.00		1.00	
Urban area	1.08	0.74–1.59	0.99	0.62–1.62	1.26	0.95–1.66	0.90	0.66–1.24
**CSI-B-related diseases**								
0 diseases	1.00		1.00		1.00		1.00	
1 or 2 diseases	11.67	6.66–20.46	3.58	1.76–7.29	8.17	5.87–11.37	3.51	2.37–5.18
**Smoking**^**b**^								
Non-smoker	1.00		1.00		1.00		1.00	
Ex-smoker	0.93	0.68–1.28	0.85	0.58–1.25	1.91	1.54–2.36	1.45	1.12–1.87
Smoker	1.14	0.74–1.76	0.90	0.54–1.48	1.93	1.40–2.66	1.34	0.93–1.95
**Alcohol intake**^**b**^								
Non-drinker	1.00		1.00		1.00		1.00	
≤ 1 day per week	0.74	0.47–1.14	0.61	0.36–1.02	1.31	1.07–1.61	1.23	0.97–1.56
> 1 day per week	0.53	0.38–0.72	0.56	0.38–0.81	0.96	0.78–1.18	0.99	0.78–1.26
**Coffee intake**^**b**^								
Non-drinker	1.00		1.00		1.00		1.00	
≤ 1 day per week	1.37	0.80–2.36	1.37	0.74–2.54	0.77	0.57–1.03	0.80	0.58–1.10
> 1 day per week	1.11	0.71–1.74	1.16	0.70–1.93	0.72	0.57–0.91	0.69	0.54–0.89
**Exercise habits**^**b**^								
No	1.00		1.00		1.00		1.00	
Yes	0.45	0.34–0.60	0.68	0.48–0.97	0.42	0.36–0.50	0.55	0.45–0.66
**Sleeping hours per day**^**b**^								
≤ 5 h	3.09	2.31–4.13	2.18	1.52–3.13	2.54	2.18–2.98	1.98	1.64–2.38
6–9 h	1.00		1.00		1.00		1.00	
≥ 10 h	2.71	0.65–11.39	4.66	0.94–23.21	1.45	0.19–10.85	1.97	0.22–17.34
**Perceived stress**^**b**^								
Low	1.00		1.00		1.00		1.00	
High	8.77	6.34–12.37	5.00	3.33–7.49	8.44	6.72–10.61	4.91	3.80–6.34
**Resilience**^**b**^								
Low	4.29	3.14–5.88	2.94	2.00–4.33	3.46	2.89–4.14	2.71	2.20–3.36
Moderate	1.00		1.00		1.00		1.00	
High	0.51	0.34–0.77	0.45	0.27–0.75	0.62	0.50–0.77	0.66	0.52–0.84

^a^cOR and aOR mean crude and adjusted odds ratios analyzed with univariate and multivariable logistic regression models.

^b^Participants with missing values for smoking (n = 494), alcohol intake (n = 1522), coffee intake (n = 3761), exercise habits (n = 458), sleeping hours per day (n = 69), perceived stress (n = 198), and resilience (n = 120) were excluded.

In the multivariable logistic regression model, CSS showed that the significantly adjusted odds ratio (aOR) (95%CI) of having 1 or 2 CSI-B-related diseases was 3.58 (1.76 to 7.29) for men and 3.51 (2.37 to 5.18) for women. Several significant factors were identified. The 80–97 yr. age group 2.07(1.06–4.02) and 2.89 (1.70–4.94), fewer sleeping hours per day 2.18 (1.52–3.13) and 1.98 (1.64–2.38), high perceived stress 5.00 (3.33–7.49) and 4.91 (3.80–6.34), and low resilience 2.94 (2.00–4.33) and 2.71 (2.20–3.36) showed higher aOR, and high resilience 0.45 (0.27–0.75) and 0.66 (0.52–0.84) and exercise habits 0.68 (0.48–0.97) and 0.55 (0.45-0.63) showed lower aOR in men and women, respectively. Furthermore, the 20–39 age group 3.13 (1.07–9.19) in men, 40–59 age group 1.95 (1.59–2.38), and ex-smokers 1.45 (1.12–1.87) in women showed higher aOR, while coffee intake (> 1 day per week) 0.69 (0.54–0.89) in women and alcohol intake (> 1 day per week) 0.56 (0.38–0.81) in men showed lower aOR (Table 3).

## Discussion

In the present study, the prevalence of CSS (CSI-A score ≥ 40) in all participants was 4.2% (95% CI 3.9 to 4.4), and was significantly higher in women than in men (4.9% vs 2.7%, *P* < 0.001). To the best of our knowledge, the present study is the first to report the prevalence of CSS in a general population.

The prevalence of CSS in the present study was 4.2% and differed with sex. The CSI-A Japanese version had higher Cronbach’s alpha and test–retest reliability than the English version: 0.879 and 0.817, and 0.89 and 0.85, respectively^8,9^. We confirmed that CSI-A in the present study was stable with Cronbach’s alpha for all participants, men, and women of 0.893, 0.889, and 0.893, respectively. Furthermore, the accuracy and specificity of a cut-off CSI-A score of 40 points were 81 and 75%, respectively, and the area under the curve was 0.86^15^. The prevalence of CSS in Japanese patients with musculoskeletal disorders was previously reported to be 11%, and the average CSI-A score was 21.9 (SD 13.3)^9^. These patients with chronic pain had a higher CSS prevalence and mean CSI-A score than our healthy population; the present results showed that the prevalence of CSS was 4.2%, and the mean CSI-A score was 15.9 (11.5) in the general population, which is considered to be appropriate. A previous study reported that the prevalence of CSS was 24.8% in healthy individuals; however, the sample size was very small^16^. We compared Japanese patients to those in other countries and found a lower mean CSI-A score in the former^8,17,18^, suggesting the possibility of racial differences.

The present results demonstrated that the prevalence of CSS was higher in healthy women than in men. In patients with musculoskeletal disorders, the prevalence of CSS (CSI-A score ≥ 40) was also higher in women than in men^19–21^. Therefore, sex may be an independent factor in the pathology of CSS.

Although rare CSI-B-related diseases were included in the present study, the main diseases associated with CSS were depression (0.79%), anxiety or panic attacks (0.24%), M/TTH (0.08%), and IBS (0.06%), which is consistent with previous findings showing high CSI-A scores in individuals with more than one CSI-B-related disease^9,16^.

The pathology of CSS has not yet been elucidated in detail; however, the mechanisms underlying pain hypersensitivity syndrome after peripheral tissue injury have been shown to involve the central sensitization of high-threshold primary afferents and increased spinal excitability^1,22,23^. The present results also indicated that participants with exercise habits and high resilience have lower CSS. In contrast, participants with high perceived stress, fewer sleeping hours per day, and low resilience have higher CSS.

Smoking has been associated with an increased risk of chronic pain development^24^. It may dysregulate homeostatic pain processes, producing an allostatic state of pain facilitation^25^. In the present study, CSS were more prevalent among female ex-smokers, which suggests that they quit smoking due to chronic pain. Due to the low smoking rate in the healthy female population, a relationship was not observed between smoking and CSS. However, this cannot explain the lack of a relationship between smoking and CSS in men. A conceivable reason was the low prevalence of CSS in the healthy male population, which was not associated with smoking, because we previously reported a relationship between CSS and smoking in a patient group^26^. Another study demonstrated that nicotine exerted temporary analgesic effects^27^; however, CSS is characterized by chronic pain, and this study found no relationship with CSS in smokers of either sex.

Previous studies also showed that moderate alcohol consumption^28^ and coffee intake^29^ alleviated pain regardless of sex. Since these were populations with a low prevalence of CSS, men who consume alcohol at least one day a week and women who drink coffee at least one day a week may mostly be healthy. The frequency of alcohol intake differs between men and women, which may be a contributing factor to the lower prevalence of CSS in men. One potential reason for the lack of a relationship between coffee intake and CSS in men may be due to the strategies used to cope with stress and other CSS-related factors in some men who drink coffee. However, these sex differences remain unclear and warrant further study.

The present results clarified the prevalence of CSS and provided basic data on CSI-A and -B based on sex and age groups in a general population using a large sample size. CSI-A is a useful tool for assessing CSS according to an international consensus. Several limitations need to be addressed. The present study included an information bias and participants may have underestimated CSI-B-related diseases because of the self-reported survey. However, agreement between self-reported CSI-B-related diseases and their diagnoses by physicians was previously reported to be high^15^, and it is a commonly used method in large-scale epidemiological studies. Furthermore, since this was a cross-sectional study, the causal relationship and sex difference between the prevalence of CSS and significant factors currently remains unclear; therefore, future longitudinal and quantitative studies on smoking, alcohol intake, and coffee consumption are needed. Another limitation is that the average age of Japanese communities is older, with fewer younger men, which needs to be considered when interpreting the present results.

In conclusion, the prevalence of CSS in the present study was 4.2% in the population examined and was higher in women than in men. CSS-related diseases and a few other factors correlated with CSS. The present results provide important information for future epidemiological research on CSS.

## Methods

### Study design and participants

A large-scale cross-sectional study was performed in Japan in the fiscal year of 2019. During the 11-month study period, we recruited all residents who were eligible for health checkups. They were from Utsunomiya city and Nasu town located approximately 100 and 150 km, respectively, northeast of Tokyo, in Tochigi prefecture. In the present study, a total of 39,152 residents were invited to complete this survey using an anonymous self-reported questionnaire when they underwent annual health check-ups. Written informed consent was provided by residents to participate according to the Declaration of Helsinki, and the Institutional Review Boards of Dokkyo Medical University approved the study protocol (No: R-7-3). A total of 24,189 out of 39,152 residents (61.8%) agreed to participate in the present study and responded to the questionnaire (Fig. 1).

### Questionnaire

Participants were instructed to complete the questionnaire, which included questions on age, sex, smoking (non-smoker, ex-smoker, and smoker), coffee and alcohol intakes (non-drinker, ≤ or > one day per week), exercise habits (walking more than one hr. per day or similar physical activity, yes, no), perceived stress (high or low), sleeping hours per day (≤ 5 h., 6–9 h., and ≥ 10 h.), and resilience (low, moderate, and high). CSS were assessed using the CSI Japanese version, comprising parts A (CSI-A) and B (CSI-B)^9^. CSI-A addresses 25 items on a 5-point Likert scale for CSS-related somatic symptoms (score, 0–100). Participants with a CSI-A score ≥ 40 were defined as having CSS^6,8,9^. CSI-B was evaluated according to 10 self-reported CSS-related diseases: RLS, CFS, FM, TJD, M/TTH, IBS, MCS, neck injury (including whiplash), anxiety or panic attacks, and depression.

### Statistical analysis

Each variable was described by sex. Age was divided into 4 groups (20–39, 40–59, 60–79, and 80–97 yr.). The reliability of CSI-A in the present study was discussed using Cronbach’s coefficient alpha. The average CSI score (standard deviation) and prevalence of CSS (CSI-A score ≥ 40 points) (95% Confidence interval, 95% CI) were analyzed using descriptive statistics by sex and age groups. The Mann–Whitney test or Kruskal–Wallis test with the Bonferroni post-hoc test was performed to assess differences in CSI scores in the sex or age groups. The 10 diseases in CSI-B were re-categorized as 0 diseases and 1 or 2 diseases. The relationships between CSS and lifestyle and mental factors were examined using the chi-squared test or Fisher’s exact test. Univariate and multivariable analyses using logistic regression models were performed to identify factors contributing to CSS in the general population. All analyses excluded missing values for each variable.

All statistical analyses were conducted using an assumed type I error rate of 0.05 with SPSS Statistics 26.0 (IBM SPSS, Inc., Tokyo, Japan).

## Data Availability

The datasets generated and analyzed during the present study are available from the corresponding author upon reasonable request.
