# Publisher Correction: A large-scale population-based epidemiological study on the prevalence of central sensitization syndromes in Japan

**DOI:** 10.1038/s41598-021-04281-w

**Published:** 2021-12-23

**Authors:** Yasuo Haruyama, Toshimi Sairenchi, Koji Uchiyama, Keisuke Suzuki, Koichi Hirata, Gen Kobashi

**Affiliations:** 1 Integrated Research Faculty for Advanced Medical Sciences, Dokkyo Medical University, 880 Kitakobayashi, Mibu, Shimotsuga, Tochigi 321-0293 Japan; 2 Department of Public Health, Dokkyo Medical University School of Medicine, Tochigi, 321-0293 Japan; 3 Laboratory of International Environmental Health, Center for International Cooperation, Dokkyo Medical University, Tochigi, 321-0293 Japan; 4 Department of Neurology, Dokkyo Medical University, Tochigi, 321-0293 Japan

Correction to: *Scientific Reports* https://doi.org/10.1038/s41598-021-02678-1, published online 02 December 2021

The original version of this Article contained an error in Affiliation 2, which was incorrectly labelled as a present address.

As a result, this affiliation was incorrectly captured for Gen Kobashi. The correct affiliations are listed below.

Integrated Research Faculty for Advanced Medical Sciences, Dokkyo Medical University, 880 Kitakobayashi, Mibu, Shimotsuga, Tochigi, 321-0293, Japan

Department of Public Health, Dokkyo Medical University School of Medicine, Tochigi, 321-0293, Japan

The original Article has been corrected.

